# Estimating the health burden of aflatoxin attributable stunting among children in low income countries of Africa

**DOI:** 10.1038/s41598-020-80356-4

**Published:** 2021-01-15

**Authors:** Hifza Rasheed, Ya Xu, Martin E. Kimanya, Xiaoxi Pan, Zhihua Li, Xiaobo Zou, Candida P. Shirima, Melvin Holmes, Michael N. Routledge, Yun Yun Gong

**Affiliations:** 1 School of Food Science and Nutrition, University of Leeds, Leeds, UK; 2 Pakistan Council of Research in Water Resources, Islamabad, Pakistan; 3 School of Medicine, University of Leeds, Leeds, LS2 9JT UK; 4 School of Life Sciences and Bioengineering, Nelson Mandela African Institution of Science and Technology, P. O. Box 447, Arusha, Tanzania; 5 School of Food and Biological Engineering, Jiangsu University, Zhenjiang, China; 6 Tanzania Bureau of Standards (TBS), P. O. Box 9524, Dar es Salaam, Tanzania

**Keywords:** Biomarkers, Risk factors

## Abstract

Numerous population-based studies have documented high prevalence of aflatoxin associated childhood stunting in low income countries. We provide an estimate of the disease burden of aflatoxin related stunting using data from the four African countries. For this empirical analysis, we obtained blood aflatoxin albumin adduct biomarker based exposure data as measured using ELISA technique and anthropometric measurement data from surveys done over a 12-year period from 2001 to 2012 in four low income countries in Africa. We used these data to calculate population attributable risk (PAR), life time disease burden for children under five by comparing two groups of stunted children using both prevalence and incidence-based approaches. We combined prevalence estimates with a disability weight, measuring childhood stunting and co-occurrence of stunting-underweight to produce years lived with disability. Using a previously reported mortality, years of life lost were estimated. We used probabilistic analysis to model these associations to estimate the disability-adjusted life-years (DALYs), and compared these with those given by the Institute for Health Metrics and Evaluation’s Global Burden of Disease (GBD) 2016 study. The PAR increased from 3 to 36% for aflatoxin-related stunting and 14–50% for co-occurrence of stunting and underweight. Using prevalence-based approach, children with aflatoxin related stunting resulted in 48,965.20 (95% uncertainty interval (UI): 45,868.75–52,207.53) DALYs per 100,000 individuals. Children with co-occurrence of stunting and underweight due to exposure to aflatoxin resulted in 40,703.41 (95% UI: 38,041.57–43,517.89) DALYs per 100,000 individuals. Uncertainty analysis revealed that reducing aflatoxin exposure in high exposure areas upto non-detectable levels could save the stunting DALYs up to 50%. The burden of childhood all causes stunting is greater in countries with higher aflatoxin exposure such as Benin. In high exposure areas, these results might help guide research protocols and prioritisation efforts and focus aflatoxin exposure reduction. HEFCE Global Challenge Research Fund Aflatoxin project.

## Introduction

Childhood stunting is considered as a main consequence of poor nutrition and multiple exposures (social and environmental etc.) affecting one-third of children under 5 and is responsible for about 15% of under 5 mortality worldwide^1,2^. Impaired child growth as a result of nutrient deficiencies, recurrent infections, exposure to fecal–oral contamination and environmental toxins poses an increased risk of morbidity and mortality from infectious diseases and impaired mental development^3^. As of 2013, more than half of all stunted children under 5 lived in Asia (56%) and more than one third lived in Africa (38%)^4^. Despite several programmes launched to prevent malnutrition, the global and regional child malnutrition estimates of the United Nations Children Education Funds^4^ from 2000 to 2016 revealed minor improvement in reducing child stunting in South Asia (38 to 24%) and Africa (38 to 31%). These are the regions of low and middle income countries (LMIC), where growth faltering has not been entirely explained by inadequate nutrition alone, whilst foodborne aflatoxin exposure especially through staple food such as maize and groundnuts is also more prevalent^5^. The dietary exposure to aflatoxin, a family of mycotoxins mainly produced by *Aspergillus flavus, Aspergillus parasiticus and Aspergillus nomius* had placed approximately 4.5 billion people at health risk in the developing countries due to aflatoxin induced liver cancer, childhood stunting, acute aflatoxicosis and modulation of the immune system^6–8^.

The most common analogs of aflatoxin found in food are aflatoxin B1 (AFB1), aflatoxin B2 (AFB2), aflatoxin G1 (AFG1), and aflatoxin G2 (AFG2), with AFB1 being the most highly toxic compound^9^. The major reactive metabolite of AFB1 in the human body i.e. exo-AFB1 8,9-epoxide, which if not detoxified, can bind to double-stranded DNA to form the promutagenic AFB1-N7-guanine adduct or, following hydrolysis to the AFB1- dihydrodiol, with proteins such as albumin^10,11^. Though childhood stunting in LMICs is a multifactorial process, a growing body of scientific evidences has highlighted an association between aflatoxin exposure and childhood stunting. Both urinary AFB1-N7-guanine and serum AFB1-lys levels are associated with dietary intake of aflatoxin and are useful for estimating the AFB1 exposure^12–14^. Using the urinary biomarkers and/or serum aflatoxin-albumin (AF-alb) adducts, the link between wide spread exposure of aflatoxin in early life and child linear growth impairment have been shown in Benin and Togo^15,16^, Gambia^17,18^, Tanzania^19^, Nigeria^20^ and Nepal^21^. These studies reported that aflatoxin exposure solely or under the influence of other risk factors affects the child growth. Pre-natal and neonatal exposure of aflatoxin have been demonstrated through Transplacental transfer^22–24^, human breast milk^25^, weaning^26^ and post-weaning foods^16^ reporting the contributory role of aflatoxin exposure in child growth impairment and life-long implications.

The underlying mechanism for aflatoxin induced growth impairment so far discovered are related to its immune modulation, reduced insulin like growth factor 1 (IGF-1), and aflatoxin-induced enteropathy^27–29^. Mupunga et al.^30^ had reported the interference of aflatoxin with intestinal integrity and hepatic metabolism thus contributing in malabsorption, micronutrient deficiencies, impaired immune function, and vulnerability to gut infections, all of which may lead to impaired growth and malnutrition. Aflatoxin was also reported to modulate immune function in vitro and in animals^31^. A small number of studies have also shown changes in immune function in humans^32^. Similarly, aflatoxin with co-exposure of fumonisin and deoxynivalenol (DON) mediate intestinal damage leading to chronic systemic immune activation and malabsorption of nutrients, and consequently to growth retardation^33,34^.

Apart from health damage caused by aflatoxin exposure, its socio-economic consequences are also crucial. Estimated economic loss may manifest in reduced learning capacity in school, chronic diseases in adulthood linked with compromised loss of working hours/days and lower earning potential and ultimately decreasing the quality of life. The Global Burden of Disease (GBD) Injuries and Risk Factors Study  2013^35^ has estimated the burden of stunting attributable to various risks, however aflatoxin exposure is so far not included as a reason of childhood stunting probably due to data paucity. Considering the availability of empirical data on prevalence of exposure to aflatoxin from past studies, the present study has attempted to evaluate the link between aflatoxin exposure as mesaured using blood aflatoxin albumin adduct biomarker using ELISA technique  (references see Table 1) and socio-economic consequences by determining the burden of child stunting in terms of DALYs as a case study in African villages.

**Table 1:** Studies included in the analysis of disease burden.

Author, year	Country	Study design	Study area	Study recruitment dates	Age range at recruitment; Visit number	No. of participants recruited	Female (%)	No. of follow up visits
Gong et al. (2004) ^16^	Benin	Cohort	Four villages (Bagbe, Sedje, Djidja, and Dovi-Cogbe)	2001	16–37 (months); 3rd	200	49	3
Gong et al. (2002); Gong et al. (2003) ^15,26^	Benin and Togo	Cross-sectional study	16 villages (30/village)	2000	9–60 (months); 1st	480	48	1
Shirima et al. (2015)^19^	Tanzania	cohort	Iringa Nyabula village, Tabora Kigwa village, and Kilimanjaro Kikelelwa village	2010–11	5–13 (months): 3rd	166	52	3
Watson et al. (2017)*^18^	The Gambia	Cohort	West Kiang district of The Gambia*	2011–12	6 months: 3rd	374	49	3

*Infants born between May 2011 and December 2012 from the Early Nutrition and Immune Development (ENID, ISRCTN49285450) trial were recruited for an aflatoxin exposure assessment.

### Study design and participants

The data used in this study originated from four previous studies on aflatoxin exposure assessment in different agro-ecological zones of western and eastern Sub-Sahara African countries, where maize and/or groundnut is predominantly produced with slight variation of harvest time. These prospective cohort studies or case–control studies were selected based on the well-defined sample sizes, aflatoxin exposure or dose assessments, anthropometric measurement and appropriate multivariate analyses. A brief detail of the selected studies is tabulated as Table 1.

**Figure 1:**
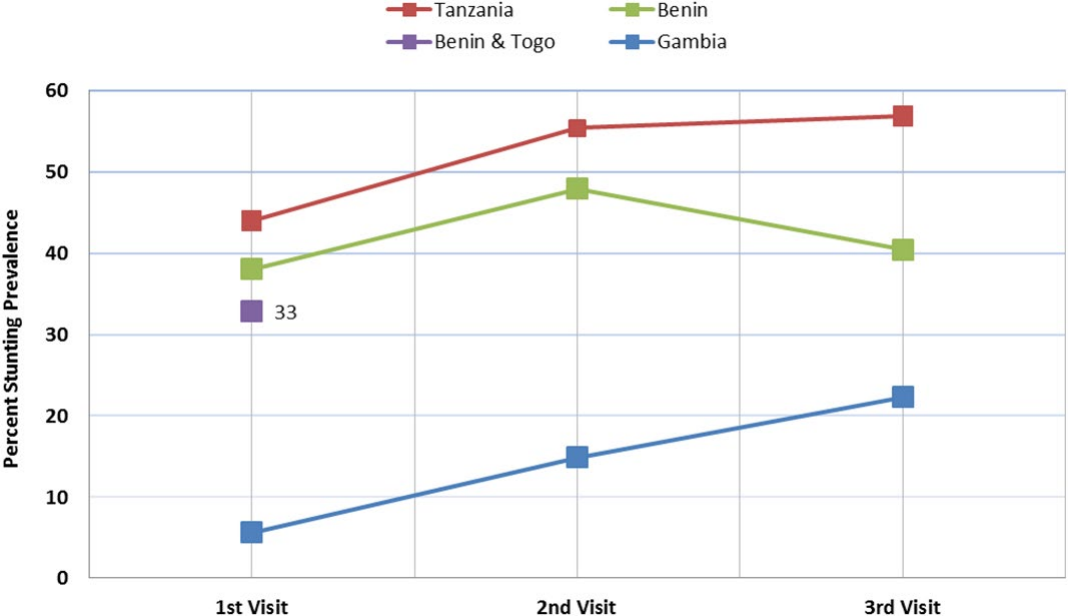
Prevalence of stunting (HAZ < − 2) in the selected study area with significant difference (*P* = 0.0005) between (HAZ > − 2) and (HAZ < − 2) of each visit for all countries.

**Figure 2:**
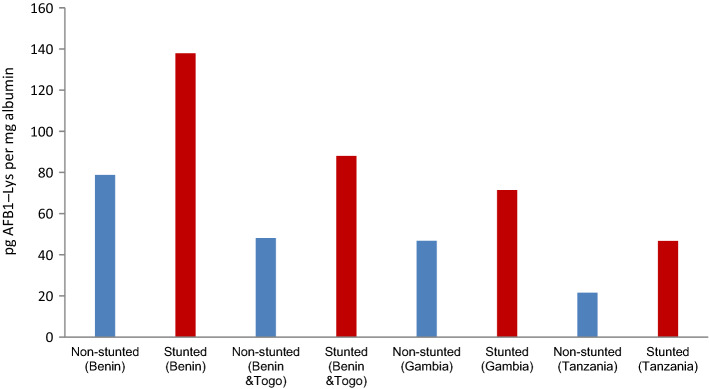
Mean conc. of AF-alb (pg/mg albumin) in stunted and non-stunted populations.

**Figure 3:**
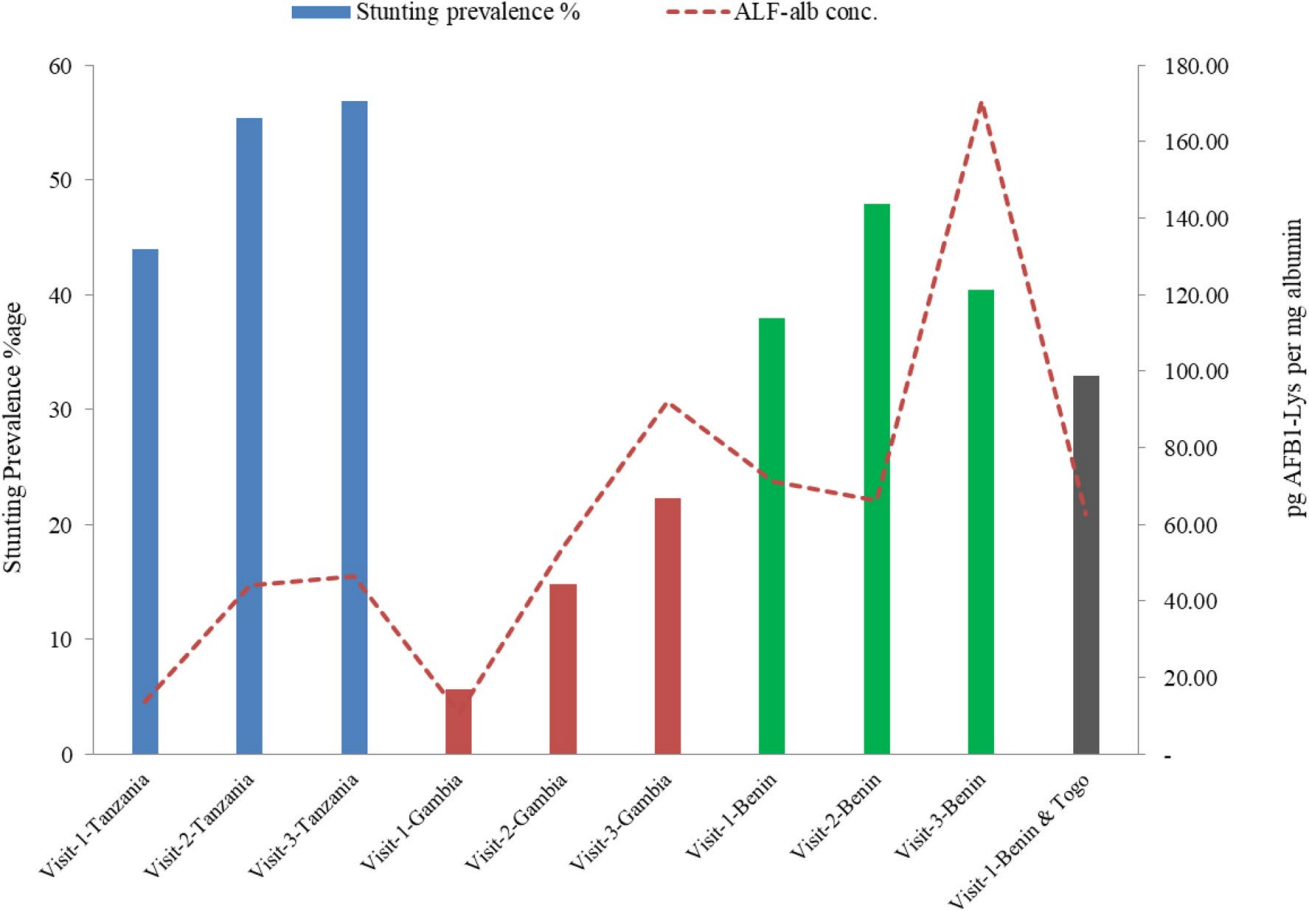
Visit wise prevalence of stunting %age vs, conc. of AF-alb (pg/mg albumin).

Considering stunting as an outcome, the study participants were characterised with respect to their stunting status as shown in Figs. 1, 2 and 3. The studies used questionnaire based interviews and 24-h dietary recall questionnaire to gather the information on child age, sex, birth weight, breast feeding, weaning age, HBV vaccination, socioeconomic status, family size or dietary consumption in one or all of the studies. These data were used in  the current analysis.

To estimate the prevalence of stunting and number of stunted children under five, this study included the children of age 5 months to under five years at recruitment. Children recruited in all four studies have been followed for aflatoxin exposure by measuring aflatoxin albumin adducts in the blood sample using an ELISA method (see Table 2 for visit wise concentrations of AF-alb concentration) and anthropometric measurements over 1 or 3 time points during maize harvest and/or storage periods.

**Table 2:** AF-alb concentration (pg/mg albumin) levels in human sera in the study areas.

Country	Overall detectable AF-alb (%)	1st Visit	2nd Visit	3rd visit
		Mean ± SD	Mean ± SD	Mean ± SD
Benin	99	71.34 ± 94.30	66.15 ± 82.17	170.51 ± 219.44
Benin and Togo	100	62.60 ± 101.08	–	–
Tanzania	91	9.52 ± 17.33	38.61 ± 61.28	39.25 ± 54.99
The Gambia	91	10.70 ± 60.91	54.07 ± 94.62	91.88 ± 130.13

These studies measured anthropometric parameters such as body height and weight, weight-for-age (WAZ), height/length-for-age (HAZ, Fig. 1) and weight-for-height/length Z-scores (WHZ) were calculated at multiple times during follow-up in all cohorts. Applying 2006 WHO Standards^36^ and the 1977 NCHS/WHO Reference^37^, stunting prevalence was calculated and reflected as in Fig. 1.

The level of aflatoxin exposure and childhood stunting in above listed four studies also indicated a consistent relationship between aflatoxin exposure and childhood growth (Figs. 2 and 3) and possibility of biologically plausibility independent of and together with other risk factors.

The logistic regression analysis between levels of AF-alb (with log and without log) and stunting status (Yes/No) was performed to see the relationship. Details of prerequisite tests for logistic regression (Supplementary material: Table 1S) revealed the significant association (*p* < 0.05) between stunting and AF-alb concentration. The DALYs for stunting and aflatoxin associated stunting were calculated, as described below.

### Calculation of all causes and cause-specific DALYs for childhood stunting

DALYs are healthy life years lost, that combines the adjusted number of years lived with disability (YLDs) and the number of years of life lost due to premature mortality (YLLs)^38^ calculated using Eqs. (1) to (3).

1 $$DALYs=YLL+YLD$$

DALYs under five for all cause stunting was calculated based on data collected from the field visits for up to age under five. DALYs for all cause stunting were also calculated from a lifetime perspective based on age specific life expectancy of each country given by the Global burden of disease study^39^.

2 $$No.\;of\;deaths\;due\;to\;stunting\; \times \;Life\;Expectancy\;at\;age\;of\;death$$

3 $$No.\;of\;stunting\;cases\; \times \;mean\;duration\;of\;disease\; \times \;disability\;weight$$

### Criteria used for calculation of YLL

The data of past studies^40–42^ have revealed higher bio-availability of aflatoxin metabolites in children relative to their body weight resulting in their limited detoxification capacity for AFB1. Knipstein, Huang^31^ have reported that growth hormone (GH) resistance occurs in children with aflatoxin induced chronic liver injury and thus GH-resistance is presented as a candidate mechanism by which AFB1 might cause stunting. Consequently, stunted and/or underweight children were observed to be significantly at higher risk of dying from infectious diseases, increased health problems, cognitive impairments, lower school achievements, reduced life-time earnings, and decreased productivity^43,44^. According to the findings of Briend et al.^45^ and Olofin et al.^46^ children with co-occurrence of stunting and underweight are considered at higher risk with increased hazards of death from diarrhoea, pneumonia, and measles with decreased *Z* scores. Likewise reported child fatalities due to AFB1 after 1–3 weeks exposure of 20 μg/kg BW/day^47^.

**Figure 4:**
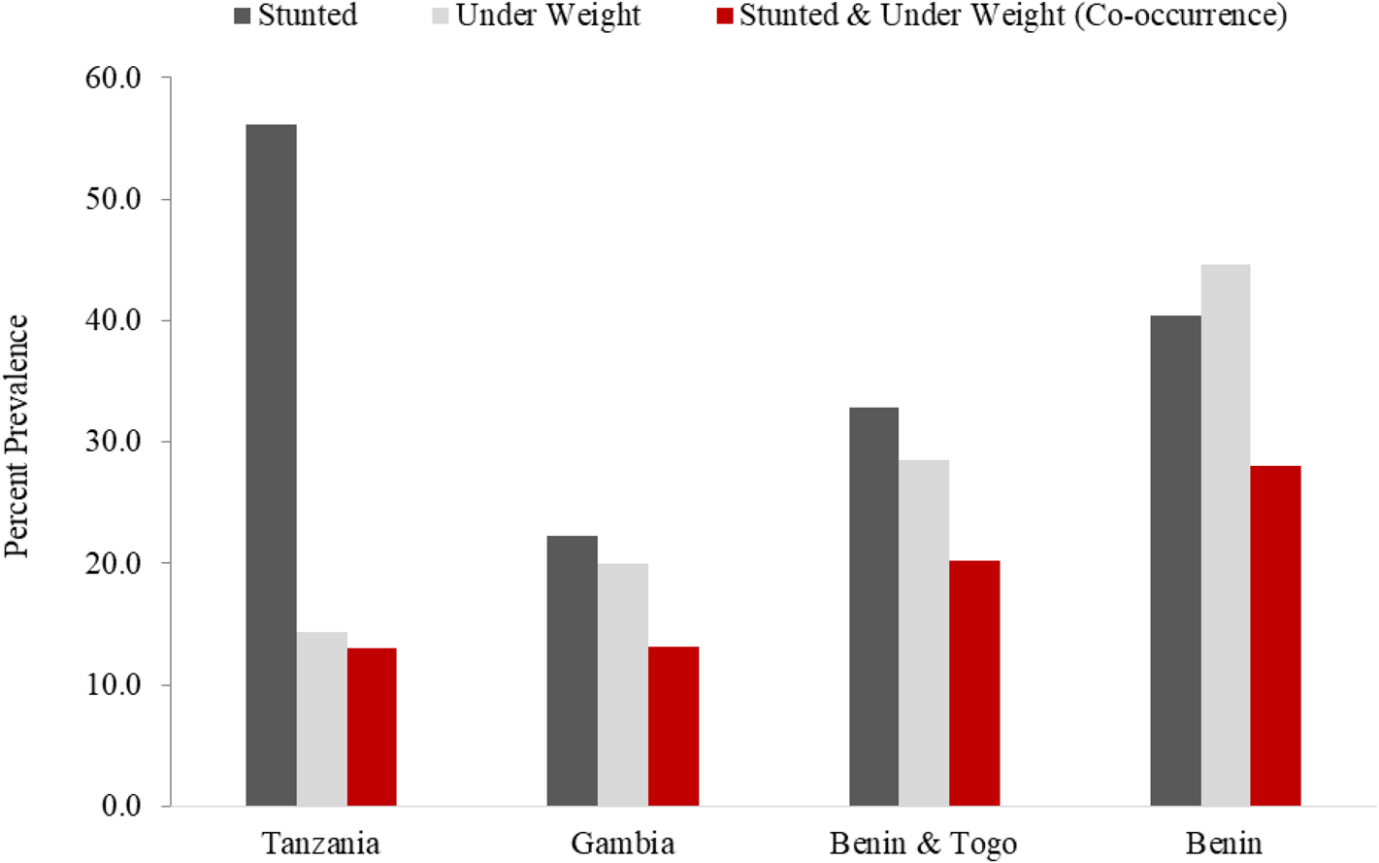
Prevalence of stunting, underweight and co-occurrence of both in study participants.

The study by Olofin et al.^46^ have determined the all-cause and cause-specific mortality hazard ratios (HR) in relation to child growth indicator ranged as 1.56 (0.98, 2.46) for HAZ (− 2 to < − 1) and 6.41(3.77, 10.89) for HAZ < − 3, whilst HR for Weight-for-Age Z score (WAZ) was 1.72 (1.08, 2.73) for WAZ (− 2 to < − 1) and 12.80 (6.97, 23.49) for WAZ < − 3. Black et al.^1^ and^48^ had estimated the deaths attributable to nutritional disorders using statistics of deaths for under five by UN Interagency Group on Mortality Estimation and prevalence estimates from the UN and Nutrition Impact Model Study (NIMS). The estimates by Black et al.^1^ for mortality of stunted (14.7%), underweight (14.4%), and wasted children (12.6%) in LMICs also confirmed the previously calculated cause specific mortality estimates by Black et al.^48^, Pelletier et al.^49^, Caulfield et al.^50^ and Olofin et al.^46^. The calculations by Black et al.^1^ for mortality risk associated with stunting and wasting were the same using different data source such as UN or NIMS prevalence estimates. Moreover, about 36.6% of children under five were reported to be stunted in sub-Saharan Africa (SSA) in 2015^35^. Compared to this high stunting prevalence, the mortality risk of stunting (age 1–4 years) reported in Global Disease Study 2016^51^ were estimated to be 0.02%, 0.05%, 0.06% and 0.05% for Togo, Gambia, Benin and Tanzania respectively. Since, the real time data produced from four selected aflatoxin studies (Table 1), children showed 23–69% of co-occurrence of stunting and underweight (Fig. 4) suggesting the possibility of higher mortality risk among stunted children. Thus, to avoid underestimation, the association of mortality with HAZ and WAZ as reported by Olofin et al.^46^ and respective mortality rates (14.7%) and (14.4%) reported by Black et al.^1^ was used to establish the assumptions for YLL calculation.

Three different models were computed for modelling the burden of stunting in these countries. Model-1 was based on the number of stunting cases (HAZ < − 2SD) compared to non-stunted children in all populations, Model-2 considered the number of children at co-occurrence of stunting and underweight (HAZ & WAZ < − 2SD) compared to those without this co-occurrence, whilst Model-3 took into account the children at co-occurrence of stunting-underweight (HAZ & WAZ < − 2SD) compared to remaining all children without this co-occurrence i.e. either stunted or under-weight or healthy children.

### Criteria used for calculation of YLD

Victora et al.^52^ reported that stunting is especially difficult to reverse after 36 months of age, whilst the *Lancet* nutrition series estimated that the nutrition-specific interventions together, if scaled up to 90%, would reduce the prevalence of stunting by only about 20%^3^. Similarly, Svefors et al.^53^ reported that children stunted at 4.5 years usually remain stunted at 5 years and later. According to International Food Policy Research Institute^54^ dietary aflatoxin exposure in the intervention group would need to be reduced by at least 35% for a detectable impact on child growth. Considering these findings, we have also assumed the possible effects of interventions such as nutritional supplements and aflatoxin exposure reduction to reverse the stunting within first 36 months of age by 20% thus saving the disability-adjusted life years (DALYs) in the sub-Sahara African countries. Considering this, we have calculated DALYs taking into account the YLL and YLD for up to 5 years based on strictly empirical data and from a lifetime perspective.

For the stunted children, YLDs were calculated as the sum of months the children had been stunted or stunted and underweight from birth to third/last visit in each study (i.e., they had a HAZ and/or HAZ &WAZ below -2 from the WHO reference median) times a disability weight of 0.002 recommended by the World Health Organization^55^. Based on the under-five mortality rates as reported by Black et al.^1^, their YLLs were calculated as number of deaths due to stunting (Model-1)/stunted and underweight (Model-2 & 3) times age, sex and country specific life expectancy at age of death provided by global burden of diseases study^39^.

Of the remaining 85.3% of the stunted children (Model-1) or 85.6% of the stunted and underweight children (Model-2 & 3), there is a probability of recovery of 20% of the mild and moderately stunted (HAZ < − 2) or stunted and underweight children (HAZ & WAZ < − 2) if interventions for nutrition, disease prevention and aflatoxin exposure reduction provided. Whilst, children who were stunted or had co-occurrence (stunted and under-weight children) at third visit were assumed to be stunted at 5 years and were assumed to carry over this stunting or co-occurrence throughout life. The remaining of the stunted (65.3%) or stunted and underweight children (65.6%) were assumed to remain stunted throughout the life and their YLDs were calculated from a lifetime perspective based on age and sex specific life expectancy.

A disability weight is a weight factor that reflects the severity of the disease on a scale from 0 (perfect health) to 1 (equivalent to death).The disability weight attributed to stunting by the global burden of disease study was 0.002 and is used in this study and reflecting the severity of a disease or condition of 0.002 on a 0–1 scale^55^. Age weighting and discount rates (social weighting) were not used in this study, in line with the 2010 GBD study^56^.

### Population attributable risk (PAR)

We calculated the AF-alb adjusted relative risk (RR) of stunting per unit change in AF-alb for 3 models. RR is calculated by dividing the incidence rate among those exposed to the high concentration of AF-alb (upper tertile of AF-alb) by the incidence rate among those exposed to the low concentration of AF-alb (lower tertile). AF-alb adjusted RRs of three models were used to calculate population attributable risk (PAR) using Eq. (4)^57^.

4 $$PAR= \frac{\sum_{i=1}^{k}{ p}_{i}({RR}_{i}-1)}{\sum_{i=0}^{k}{ p}_{i}\left({RR}_{i}-1\right)+1}$$

where *pi* is the prevalence of exposure level *i*, RR is the relative risk of disease in exposure level *i* of AF-alb, and *k* is the total number of exposure levels. The PAR were then applied to the DALYs calculated for aflatoxin induced stunting. The estimated PAR quantifies the independent effect of aflatoxin on stunting (holding all other risk factors of stunting such as health status, nutritional intake, food quality, poor sanitation, and general poverty as constant).

### Sensitivity analysis

Using the probabilistic approach all YLL, YLD and DALY calculations are presented with 95% uncertainty intervals (95%UI) calculated using the @RISK software package, Version 7.5 (2018 Palisade Corp. USA)^58^. This included a 10,000-trial Monte Carlo simulation with all inputs varied simultaneously. To quantify uncertainty, we took ± 10% variation of these parameters i.e. YLL, YLD, DALY, PAR (%), thus generating 95th percentiles. To generate the 95% UI, these parameters were assumed to be normally distributed. However, following the GBD’s uncertainty principle in the absence of data and a method that would allow one to estimate the correlation of uncertainty between YLLs and YLDs, we also assumed that, for estimating DALYs, YLL and YLD uncertainty distributions were independent.

## Results

### Study population

There were 1220 children altogether enrolled in the four studies. Subjected to the availability of anthropometry record and concentration of AF-alb of 1145 children, comparison of three groups i.e. children with stunting, children with no stunting and those at co-occurrence of stunting and underweight is given at Table 3. Overall the children with stunting (109.41 pg/mg) and those at co-occurrence of stunting and underweight (132.27 pg/mg) showed higher AF-alb concentration compared to those found without stunting (75.01 pg/mg). Stunted children in Benin study and in Benin and Togo study showed a higher aflatoxin exposure in all cases. Moreover, the significant association (*p* > 0.05) between AF-alb and stunting (supplementary material: Table 1S) also convinced us to estimate DALYs.

**Table 3:** Distribution of level of AF-alb concentration (pg/mg albumin) with respect to stunted and non-stunted cases.

Study	*Total study population	Children with stunting (HAZ < − 2)			Children at co-occurrence (HAZ and WAZ < − 2)			Children with no stunting (HAZ > − 2)		
		No	%	AF-alb conc. pg/mg (95%CI)	No	%	AF-alb conc. pg/mg (95%CI)	No	%	AF-alb conc. pg/mg (95%CI)
Tanzania	146	82	56.2	52.06 (23.31–80.81)	19	13.0	31.57 (13.24–49.91)	63	43.2	40.01 (27.15–52.867)
Gambia	350	76	21.7	88.11 (67.03–109.18)	45	12.9	76.88 (57.19–96.56)	272	77.7	91.95 (74.48–109.41)
Benin & Togo	456	149	33.0	88.00 (65.28–110.73)	92	20.2	106.69 (72.49–140.90)	306	67.0	48.08 (41.09–55.15)
Benin	193	76	39.4	229.95 (175.02–284.87)	53	28.0	250.48 (182.94–318.01)	115	59.6	130.15 (94.634–165.66)
Overall	1145	383	33.5	109.41 (92.04–126.77)	209	18.3	132.37 (106.31–158.43)	756	66.0	75.01 (66.04–83.97)

*Overall population is total population is 1220, HAZ score of 75 could not be available, hence analysis is performed for 1145 participants.

RR > 1 for the three models denotes a larger incidence in the exposed than in the non-exposed indicating that exposure to the factor (AF-alb conc.) seems to increase the probability of developing the stunting (Supplementary Information: Figure-1S). The PAR% of all the three models was highest for the study population of Benin and lowest for Tanzania subjected to the AF-alb concentration (Table 4).

**Table 4:** Population attributable risk percent (PAR%) calculated for three risk models.

Country	Model-1	Model-2	Model-3
	PAR% (95% UI) for HAZ < − 2	PAR% (95% UI) for HAZ & WAZ < − 2	Remaining all including either stunted OR underweight OR normal)
Tanzania	3.04 (2.78–3.30)	14.29 (13.05–15.53)	15.16 (13.83–16.48)
Gambia	23.44 (21.41–25.48)	27.26 (24.91–29.61)	27.25 (24.90–29.62)
Benin & Togo	23.35 (21.33–25.38)	30.70 (28.04–33.38)	33.52 (30.64–36.45)
Benin	35.69 (32.58–38.77)	49.79 (45.51–54.10)	52.62 (48.08–57.20)

### YLLs, YLDs and DALYs of all causes stunting

The output measures as YLL, YLD and DALYs calculated from a lifetime perspective for models 1 and 2, are presented with 95% uncertainty intervals (95%UI) in Tables 4 and 5. The total burden of all cause stunting (model-1) was highest in Tanzania with DALYs of 366,118.51 (95% UI: 342,672.97, 389,353.08) followed by 279,297.50 (95% UI: 261,792.06, 296,843.87) DALYs in Benin. Whilst, stunting due to aflatoxin exposure caused loss of 3–36% of DALYs, highest in Benin such as 99,693.32 (95% UI: 89,271.91, 110,617.71) DALYs per 100,000 people followed by 47,783.70 (95% UI: 42,762.95,53,029.93) per 100,000 people in Benin & Togo study (Table 5).

**Table 5:** YLLs, YLDs and DALYs of stunted children.

MODEL-1	All causes stunting				Aflatoxin induced DALYs per 100,000 (95% UI)
	YLD (95% UI)	YLL (95% UI)	DALYs (95% UI)	All DALYs per 100,000 (95% UI)	
Tanzania	6.23 (5.83, 6.63)	527.92 (493.50–561.93)	534.15 (499.82–568.15)	366,118.51 (342,672.97–389,353.08)	11,143.48 (9937.60–12,388.47)
Gambia	6.07 (5.69, 6.46)	549.19 (513.80–584.25)	555.26 (519.90–590.30)	159,051.70 (148,925.49 -169,141.44)	37,283.05 (33,345.64–41,338.40)
Benin + Togo	11.45 (10.72,12.17)	917.28 (859.24–975.43)	928.73 (870.64–986.95)	204,631.51 (191,829.06–217,471.17)	47,783.70 (42,762.95–53,029.93)
Benin	5.91 (5.53, 6.28)	532.41 (498.72–566.26)	538.31 (504.62–572.13)	279,297.50 (261,792.06–296,843.87)	99,693.32 (89,271.91–110,617.71)
Overall	29.65 (28.65, 30.64)	2526.39 (2442.62–2609.54)	2556.05 (2472.29–2639.21)	252,252.45 (243,853.94–260,600.65)	48,965.20 (45,868.75–52,207.53)

DALYs per 100,000 people from all cause co-occurrence (stunting and underweight) was 176,663.83 (95% UI: 165,280.40–187,900.65) highest in Benin followed by 130,499.25 (95% UI: 122,302.17–138,858.37) in Benin & Togo. With similar pattern, aflatoxin exposure also caused 87,963.94 (95% UI: 78,457.86–97,815.26) DALYs lost due to co-occurrence (stunting and underweight) in Benin (Table 6).

**Table 6:** YLLs, YLDs and DALYs of children with co-occurrence of stunting and under-weight.

Model 2	DALYs Relative to the existing number of stunting cases			DALYs per 100,000 (95%UI)	AFB-1 DALYs per 100,000 (95%UI)
	YLD (95%UI)	YLL (95%UI)	DALYs (95%UI)		
Tanzania	1.46 (1.37–1.56)	131.99 (123.58 -140.39)	133.46 (125.04–141.85)	91,469.72 (85,697.03–97,222.87)	13,072.21 (11,678.44–14,547.19)
Gambia	3.72 (3.48–3.96)	274.48 (256.97–292.14)	278.20 (260.71–295.86)	79,685.48 (74,680.81–84,739.76)	21,719.92 (19,444.06–24,099.26)
Benin & Togo	6.98 (6.53–7.42)	587.74 (550.49–625.82)	594.72 (557.47–632.84)	130,499.25 (122,302.17–138,858.37)	40,064.74 (35,826.88–44,475.13)
Benin	4.16 (3.90–4.43)	334.61 (312.86–356.08)	338.77 (316.96–360.25)	176,663.83 (165,280.40–187,900.65)	87,963.94 (78,457.86–97,815.26)
Overall	16.33 (15.75–16.90)	1328.31 (1281.29–1375.55)	1344.63 (1297.67–1391.95)	119,550.88 (115,632.46–123,485.27)	40,703.41 (38,041.57–43,517.89)

In model-3, children with co-occurrence of stunting and under-weight in the four studies were compared with remaining all children. The difference of YLDs, YLLs and DALYs between model-3 and model-2 is negligible i.e. 176,558.47 (95% UI: 165,298.76–188,016.46) all cause DALYs lost in Benin followed by 130,543.71 (95% UI: 122,369.89–138,750.59) in Benin & Togo participants. Similar trend was found for aflatoxin induced DALYs with no considerable difference between results of models 2 (Table 6) and 3 (Supplementary information: Table 2S).

Children with stunting only (model-1) have shown comparatively higher all causes stunting DALYs with difference of 25% (Tanzania), 50% (Gambia), 64% (Benin and Togo), 63% (Benin) than children at co-occurrence of stunting and underweight (model-2) (Tables 5 & 6). Whereas, Aflatoxin induced DALYs difference between models-1 and 2 ranges between 58 to 88%. There is not much difference between DALYs of models 2 and 3. The total burden of aflatoxin caused stunting using 3 models was estimated at 48,965.20 (95% UI: 45,868.75–52,207.53) (model-1), 40,703.41 (95% UI: 38,041.57–43,517.89) (model-2) and 43,072.67 (95% UI: 40,164.58–46,054.89) (model-3) per 100,000 population.

**Figure 5:**
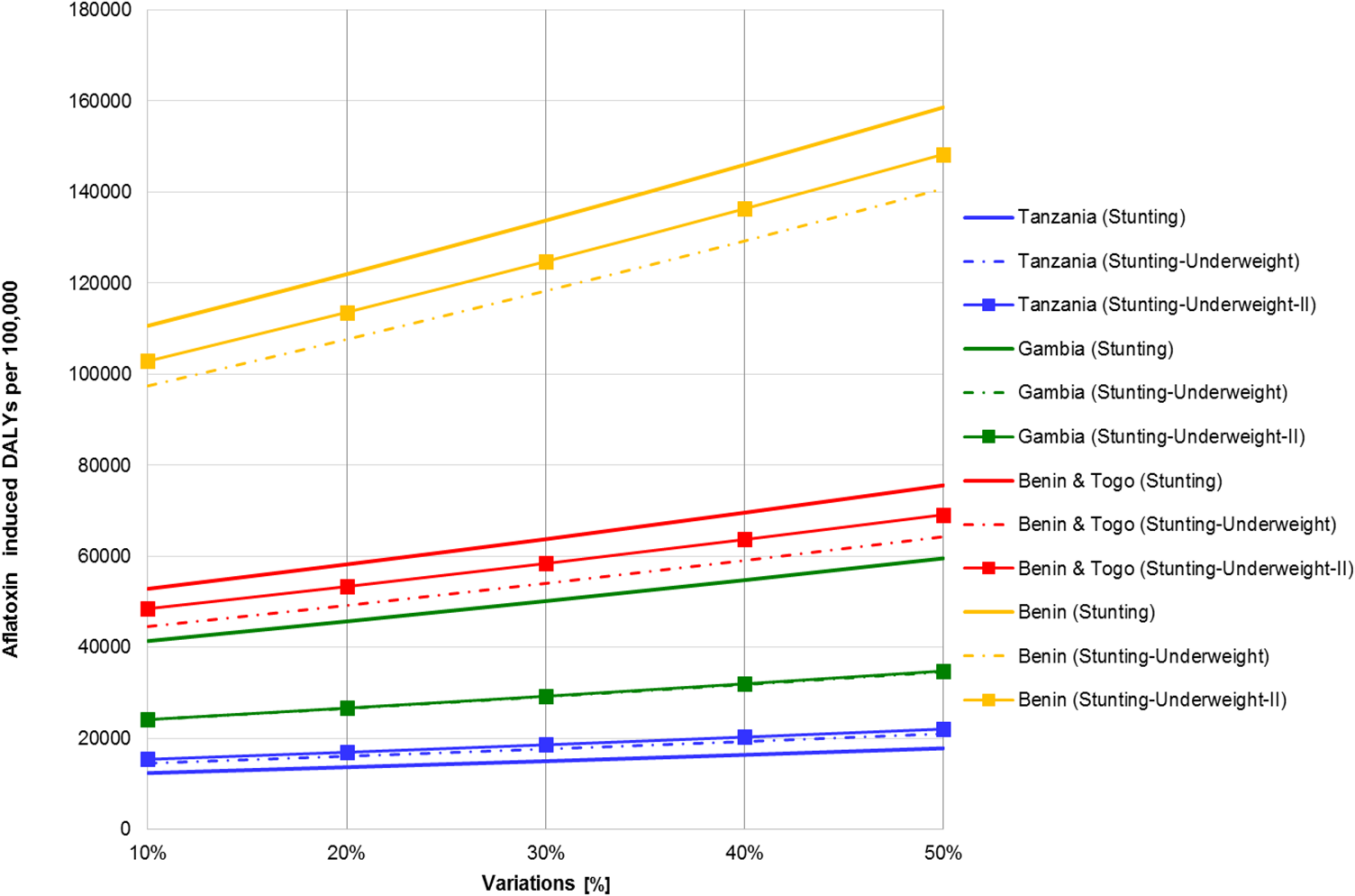
Sensitivity Analysis of Aflatoxin caused Stunting DALYs (models 1, 2 & 3).

Changing the mean (baseline) input values for YLLs and YLDs by 10–50%, the standard error on the mean for these samples analysed led to a change up to 70% for the mean output DALYs (Fig. 5).

## Discussion

Aflatoxin is a known human liver carcinogen classified by the IARC^9^. The WHO has reported the health burden of aflatoxin based on its carcinogenicity^59^. More recently, increasing evidence supports that aflatoxin may contribute to child stunting adversely^31,60,61^. Owning to the complexity in the causation of child stunting, adverse effect on child stunting by aflatoxin is not easily defined or clearly quantified. It became more widely recognised that nutrition specific intervention has not reduced child stunting as effectively as expected. Nutrition sensitive approaches extending effects to environment, food system and societal aspects are being promoted in tackling child malnutrition. In this regard, it is timely to include common hazards in the population such as aflatoxin in the intervention package. Characterising aflatoxin impact on child stunting has become an urgent matter. Following this, the current study has estimated the burden of stunting among children under five (age up to 45 months at third visit) that may be attributable to aflatoxin exposure in four African countries. The Global Burden of Disease (GBD), Injuries, and Risk Factor study 2013^62^ was the first of a series of annual updates with appropriate counterfactual risk distribution for six new risk factors including childhood stunting and wasting^62^. However, aflatoxin as a cause of this risk factor (childhood stunting) has not been included in the last Global Burden of Disease Injuries, and Risk Factor study^35,51^. Since several studies have provided the evidences of association between aflatoxin exposure and childhood stunting and bridged the data gap constraining the estimation of aflatoxin disease burden, this study is probably the first analysis that has attempted to use DALYs to measure the burden of stunting due to aflatoxin exposure in different African countries populations.

Our results suggest that 3–36% of stunting related DALYs in these populations is attributable to aflatoxin exposure. For children with both stunting and underweight, the contribution of aflatoxin exposure to lost DALYs within this sub set of children is larger (14–49%), although overall DALYs from co-occurrence is lower as this is a smaller sub set of children.

When comparing stunting burden by countries, the highest burden of stunting in different western and eastern Sub-Saharan African countries in this assessment is supported by previous prevalence studies^18,20^ reporting high prevalence of aflatoxin exposure in areas of low income countries consuming maize and ground nuts as staple food, and where storage of these facilitates the aflatoxin exposure under warm climate. Consequently, the burden of aflatoxin caused possible stunting over the human lifespan has differing patterns in regions with high or low aflatoxin exposure. Benin was the country with higher prevalence of stunting (40%) and underweight (45%) with co-occurrence of both as 28%. The prevalence of stunting and underweight children in the Gambia (22% vs 20%), Benin and Togo studies (33% vs 29%) was closely matching, however in Tanzania the difference is high: 56.2% stunting vs 14.4% underweight and co-occurrence of both is only 13%. Moreover, AFB-1 concentration and stunting prevalence in the third follow up visit compared to first and second visits forecasts the DALYs burdens to increase substantially during adulthood possibly due to increased exposure to aflatoxin through consumption of family food.

The disease burden of aflatoxin induced stunting estimated by models 1, 2 and 3 suggests that package of interventions such as nutritional interventions, health interventions, WASH interventions to reduce childhood stunting should also include the aflatoxin exposure reduction interventions with priority focus on stunted children and those affected both by stunting and underweight.

The all causes stunting DALYs in this risk assessment when compared with the 2016 Global Burden of Diseases (GBD) shows that various theoretical and methodological challenges may affect both the calculation and interpretation of DALYs estimates. The countries included in the current study (Tanzania, Benin, Togo, Gambia) are in the GBD study 2016^39^ list of countries without vital registration for 1980–2016. For such regions with missing health data, estimates are derived from other similar regions, and predictive covariates. For instance in Africa, many countries lack reliable cause specific mortality data and GBD modelling outputs may be over-reliant on inherent assumptions^63^. In GBD study (2016) stunting DALYs of four countries (9466.713 per 100,000 populations) is lower than that estimated in this analysis (729,801.72 per 100,000 populations). This difference is due to a) difference of stunting related mortality rates as in GBD, 2016^35,51^ stunting linked mortality rates of 0.02%, 0.05%, 0.06% and 0.05% for Togo, Gambia, Benin and Tanzania respectively were used which resulted in GBD assumption that stunting has very low mortality (YLL) estimation, and; b) difference in age and gender specific life expectancy i.e. country specific life expectancy of 64–68 years used in this study whilst GBD study^39^ has used the maximum life expectancy of 82 years (female) and 84 years (male) for all the countries. Comparing the stunting burden by sex indicated no considerable difference of DALYs (data not shown).

Considering the availability of adequate AFB1 exposure data, the study outcomes suggest that the GBD analysis data on childhood stunting may also include impaired child growth due to aflatoxin exposure which is causally related to childhood stunting. Moreover, there is a consistency of the DALYs burden from stunting in GBD studies (2013 to 2016) and demands the availability of high-quality data on mortality due to stunting and aflatoxin induced stunting to enact local, national, and global change for stunting reduction especially for economically disadvantaged populations.

DALYs of men were found 1.5 times higher than the DALYs in women in this study and thus there was no considerable difference in terms of gender disparity due to lower difference of stunting prevalence (data not shown). However, there may be some factors which might have influenced this disease burden analysis and cannot be ignored as a source of uncertainty for future DALY’s estimation. These include lack of control group (unexposed to AFB1), breast feeding, wean age, birth weight, socioeconomic status, co-exposure of other mycotoxins such as fumonisins and variation of time/season of 3^rd^ visits in four countries, variation in crop harvest time of maize and/or ground nut. Holding all these as constant, a 10–50 folds increase in input parameters resulted in change in DALYs up to 70% from the mean.

## Conclusions

We have analysed data from four studies that explored the association between aflatoxin exposure and child stunting. We found that aflatoxin exposure made a significant contribution to DALYs lost due to stunting, with an average of 16% of lost DALYs attributable to aflatoxin exposure. For children with both stunting and underweight, this figure was 34%. The regional heterogeneity observed in this study highlights the importance of understanding local burden of disease. The estimations provided in this study might help to address aflatoxin exposure and ultimately reduce its impact on the health of the African population.

## Supplementary Information

Supplementary Information.
