# Supplementary material for: Estimating the health burden of aflatoxin attributable stunting among children in low income countries of Africa
Source: Sci Rep. 2021 Jan 15;11:1619. doi: 10.1038/s41598-020-80356-4 (PMC7810982; doi:10.1038/s41598-020-80356-4)
Supplement: Supplementary file 1 — Supplementary Information. [file 41598_2020_80356_MOESM1_ESM.docx]

**Estimating the health burden of aflatoxin attributable stunting among children in low income countries of Africa**

Hifza Rasheed^1,2^, Ya Xu^3^, Martin E. Kimanya^4^, Xiaoxi Pan^3^, Zhihua Li^5^, Xiaobo Zou^5^, Candida P. Shirima^6^, Melvin Holmes^1^, Michael N. Routledge^3,5*^, Yun Yun Gong^1*^

^1^School of Food Science and Nutrition, University of Leeds, UK

^2^Pakistan Council of Research in Water Resources, Islamabad, Pakistan

^3^School of Medicine, University of Leeds, Leeds, LS2 9JT, UK

^4^ School of Life Sciences and Bioengineering, Nelson Mandela African Institution of Science and Technology, P. O. Box 447, Arusha, Tanzania

^5^ School of Food and Biological Engineering, Jiangsu University, Zhenjiang, China

^6^ Tanzania Bureau of Standards (TBS), P. O. Box 9524, Dar es Salaam, Tanzania

*joint corresponding authors email: [medyg@leeds.ac.uk](mailto:medyg@leeds.ac.uk); [medmnr@leeds.ac.uk](mailto:medmnr@leeds.ac.uk)

**Supplementary Material**

**Table-1S: The logistic regression analysis between levels of AF-alb (with log and without log) and stunting status (Yes/No)**

| **Cases** | **Description** | **p-value (Desired)** | **Visit (No.)** | | |
| --- | --- | --- | --- | --- | --- |
|  |  |  | **1** | **2** | **3** |
| **1** | **Stunting No/Yes and Log (AF-alb)** |  |  |  |  |
|  | Omnibus Test of Model Significance | p<0.05 | 0.00 | 0.050 | 0.170 |
|  | Hosmer & Lemeshow Fitness Test | p>0.05 | 0.00 | 0.000 | 0.018 |
|  | Wald Significance - Regression Equation | p<0.05 | 0.00 | 0.052 | 0.170 |
| **2** | **Stunting No/Yes and AF-alb** |  |  |  |  |
|  | Omnibus Test of Model Significance | p<0.05 | 0.00 | 0.040 | 0.024 |
|  | Hosmer & Lemeshow Fitness Test | p>0.05 | 0.00 | 0.001 | 0.088 |
|  | Wald Significance - Regression Equation | p<0.05 | 0.00 | 0.005 | 0.026 |
| **3** | **Stunting No/Yes and AF-alb <800** |  |  |  |  |
|  | Omnibus Test of Model Significance | p<0.05 | 0.00 | 0.010 | 0.018 |
|  | Hosmer & Lemeshow Fitness Test | p>0.05 | 0.00 | 0.001 | 0.030 |
|  | Wald Significance - Regression Equation | p<0.05 | 0.00 | 0.011 | 0.018 |

Case 1: With Log (AF-alb) and Stunting Status: No visit passes all tests, so we cannot establish regression equation.

Case 2: With AF-alb (No log) and Stunting Status: 3rd visit passes all tests, we can establish regression equation.

Case 3: With AF-alb (Values filtered to remove extreme value effect) and Stunting Status: 3rd visit passes nearly all tests, we can establish regression equation.

**
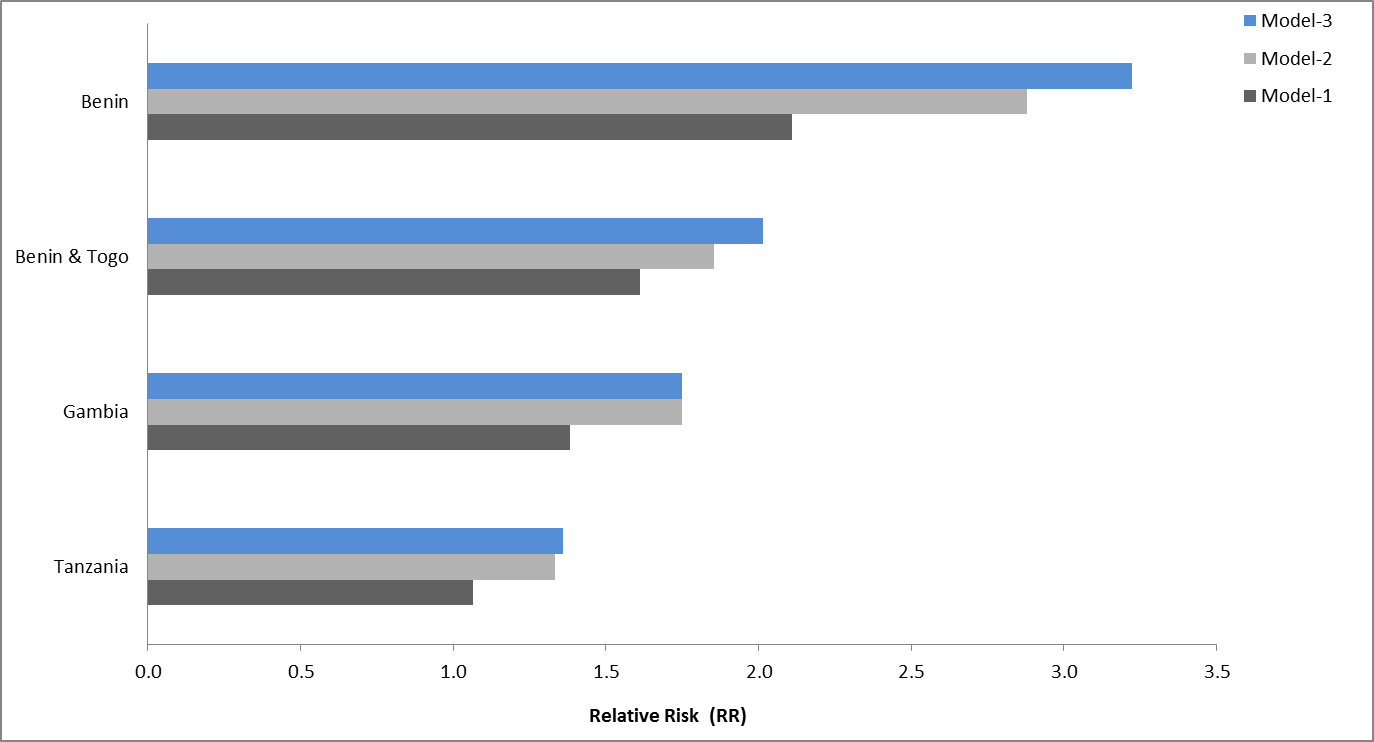
**

**Figure-1S:** AF-alb adjusted relative risk (RR) of stunting for 3 models

**Table-2S:** **YLLs, YLDs and DALYs of children with co-occurrence of stunting and under-weight compared with remaining all (Model-3)**

| **Model 3:** | **DALYs Relative to the existing number of stunting-underweight cases** | | | **DALYs per 100,000 (95%UI)** | **AFB-1 DALYs per 100,000 (95%UI)** |
| --- | --- | --- | --- | --- | --- |
|  | **YLD (95%UI)** | **YLL (95%UI)** | **DALYs (95%UI)** |  |  |
| Tanzania | 1.46  (1.37 -1.56) | 132.02  (123.60 140.37) | 133.48  (125.06 141.84) | 91,484.21  (85742.58 97210.85) | 13,867.20  (12381.04 15426.50) |
| Gambia | 3.72  (3.48 -3.96) | 274.45  (257.00 291.70) | 278.16  (260.73 295.46) | 79,675.40  (74671.47 84637.51) | 21,711.64 (19388.18 24145.04) |
| Benin & Togo | 6.98  (6.54 -7.43) | 587.94  (550.66 625.47) | 594.93  (557.66 632.40) | 130,543.71  (122369.89 138750.59) | 43,762.20  (39130.75 48584.83) |
| Benin | 4.17  (3.90 4.43) | 334.41  (312.84 356.37) | 338.57  (317.02 360.49) | 176,558.47  (165298.76 188016.46) | 92,912.69  (82904.26 103467.51) |
| Overall | 16.33  (15.76 16.91) | 1328.80  (1281.26 1376.36) | 1345.13  (1297.47 1392.54) | 119,564.57  (115587.88 123617.47) | 43,072.67  (40164.58 46054.89) |
